# AI-based multimodal fusion for preoperative prediction of breast microcalcifications: combining mammography and tomosynthesis

**DOI:** 10.1186/s12880-026-02673-w

**Published:** 2026-08-12

**Authors:** Junting Wei, Jie Li, Ruixin Pan, Mingna Cao, Chengcheng Ma, Zongyu Xie, Yichuan Ma, Zhizhen Gao

**Affiliations:** 1 School of Medical Imaging, Bengbu Medical University, Bengbu, China; 2 Department of Radiology, The First Affiliated Hospital of Bengbu Medical University, Bengbu, China; 3 Anhui Key Laboratory of Digital Medicine and Intelligent Health, Bengbu Medical University, Bengbu, China

**Keywords:** Microcalcifications, Mammography, Digital breast tomosynthesis, Radiomics, Deep learning

## Abstract

**Background:**

This study aimed to develop artificial intelligence (AI) models based on mammography (MG) and digital breast tomosynthesis (DBT) for non-invasive preoperative differentiation of benign and malignant breast microcalcifications.

**Methods:**

We retrospectively analyzed 190 patients with pathologically confirmed breast microcalcifications, who were divided into training and test sets at a ratio of 7:3. Radiomics features and deep learning scores extracted using ResNet-50 were derived from MG and DBT images. After feature selection procedures, single-modality models and an integrated model (MD model) were constructed.

**Results:**

The area under the curves (AUCs) of the BI-RADS model, DBT model, MG model, and MD model in the training set were 0.805, 0.879, 0.896, and 0.939. In the test set, the corresponding AUCs were 0.809, 0.768, 0.793, and 0.835, respectively. The MD model demonstrated significantly better diagnostic performance compared with the other models (DeLong test, *P* < 0.05). SHapley Additive exPlanations (SHAP) analysis revealed that the deep learning scores (DL scores) from the MG craniocaudal (CC) view and radiomics features reflecting textural complexity and spatial heterogeneity were the most influential features in model prediction.

**Conclusions:**

The MD model integrating radiomics and deep learning scores from MG and DBT improved the diagnostic accuracy for differentiating benign and malignant breast microcalcifications, outperforming traditional methods. It provided a valuable non-invasive preoperative tool with the potential to optimize clinical decision-making and reduce unnecessary invasive procedures.

## Background

Cancer is a major social, public health, and economic challenge in the 21st century. Breast cancer is the leading cause of cancer-related mortality among women in 112 countries [1]. Therefore, early and precise diagnosis is essential to improve patient outcomes. Compared with other imaging modalities such as ultrasound (US) and magnetic resonance imaging (MRI), mammography (MG) offers higher sensitivity for detecting breast microcalcifications and thus serves as the primary imaging modality for breast cancer screening [2, 3]. Microcalcifications are calcium salt deposits with a diameter of less than 1 mm that accumulate in breast tissue [4]. Approximately 50% of early-stage breast cancers without a palpable mass are detected by MG through the signs of microcalcifications [5, 6]. This highlights their importance in early detection and diagnosis [4, 7]. Clinicians typically classify microcalcifications according to the Breast Imaging Reporting and Data System (BI-RADS) based on their morphology, size, distribution, and associated imaging findings [8]. Although their clinical importance is well recognized, distinguishing benign from malignant microcalcifications remains challenging. Pathological biopsy is considered the gold standard for diagnosis. However, as an invasive procedure, it inevitably subjects some patients with benign lesions to unnecessary physical harm and psychological burden. Previous studies reported that approximately 60% of patients with suspicious microcalcifications are confirmed as benign after biopsy [9, 10]. Therefore, there is a strong need to develop an accurate and non-invasive method for preoperative prediction to optimize clinical decision-making and avoid over-treatment.

In recent years, artificial intelligence (AI) has rapidly advanced in the field of medical imaging. Radiomics extracts high-throughput quantitative features from medical images, such as texture, shape, and intensity. It can capture biological information that is often difficult for the human eye to perceive [11–13]. Deep learning (DL), based on architectures such as convolutional neural networks, can automatically learn discriminative imaging features. It enables end-to-end high-accuracy image classification and analysis [14, 15]. In breast cancer research, both radiomics and deep learning have demonstrated considerable value in assisting diagnosis. They have been applied to treatment response assessment, molecular subtype prediction, biomarker status, axillary lymph node status assessment, and metastatic burden evaluation [16, 17].

However, existing studies have predominantly focused on single-modality models based on either radiomics or DL features. Multimodal frameworks that integrate MG and digital breast tomosynthesis (DBT) with complementary radiomics and DL representations remain relatively limited.

In this study, we further incorporated the 3D advantages of DBT in addition to MG. We also considered the spatial characteristics of microcalcification clusters. Radiomics and DL methods were used to extract complementary features to build a more comprehensive predictive model and provide feature visualization for model interpretation. This study aimed to provide a more accurate and practical non-invasive preoperative tool for distinguishing benign from malignant breast microcalcifications. It may improve clinical decision-making and reduce unnecessary invasive procedures.

## Methods

### Study population

**Fig. 1:**
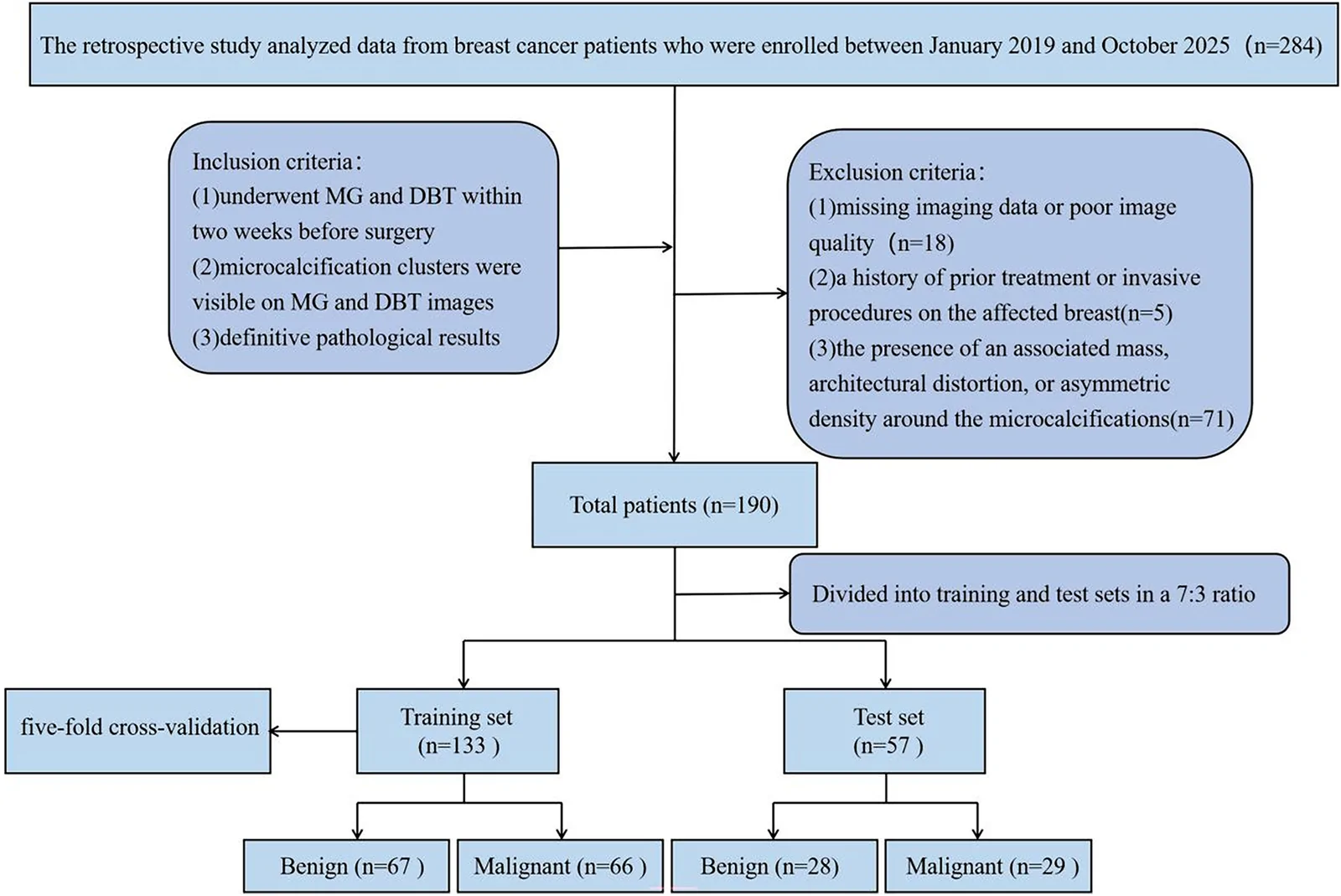
Flowchart of patient screening and dataset partitioning for the cohort with breast microcalcifications. All 190 patients were randomly divided into a training set (*n* = 133) and a test set (*n* = 57). Five-fold cross-validation was performed within the training set to evaluate model stability. The results were used as internal validation performance metrics

This retrospective study included 190 female patients with breast microcalcifications at the First Affiliated Hospital of Bengbu Medical University from January 2019 to October 2025, with postoperative pathological diagnoses serving as the gold standard. The patients were randomly divided into training and test sets at a ratio of 7:3. The inclusion criteria were as follows: (1) patients underwent MG and DBT within two weeks before surgery; (2) microcalcification clusters were visible on MG and DBT images; and (3) definitive pathological results were available. The exclusion criteria were as follows: (1) missing imaging data or poor image quality; (2) a history of treatment or invasive procedures on the affected breast; and (3) the presence of a mass, architectural distortion, or asymmetric density associated with the microcalcifications (Fig. 1). All data were retrospectively collected from clinical imaging records. Patient information was anonymized and de-identified before data analysis. The study was approved by the institutional ethics committee, and the requirement for informed consent was waived.

### Image acquisition

**Fig. 2:**
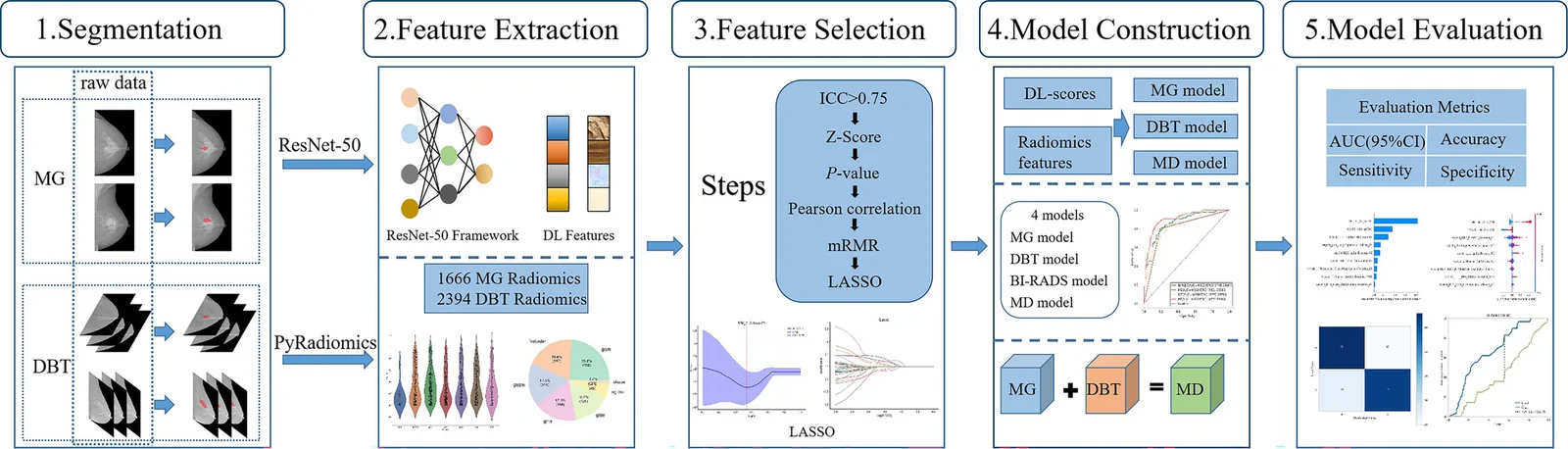
Schematic diagram of the study design and workflow

All images were acquired using a Siemens MAMMOMAT Inspiration full-field digital mammography system (Siemens Healthcare GmbH, Erlangen, Germany). Images were acquired in OPDOSE mode to obtain standard bilateral craniocaudal (CC) and mediolateral oblique (MLO) views. During DBT acquisition, predefined imaging parameters were used. The X-ray tube rotated within a ± 25° arc, and projection images were acquired at 2° angular intervals. Standard low-dose exposure settings were applied during scanning. All DBT images were reconstructed on the same workstation with a standardized reconstruction algorithm. This process generated a series of high-resolution tomosynthesis slices with a 1-mm slice thickness. All images were saved in DICOM format. The experimental design and procedure of this study are presented in Fig. 2.

### BI-RADS assessment

Two radiologists with more than five years of breast imaging experience independently assessed breast microcalcifications. All lesions were classified into categories 3, 4A, 4B, 4C, and 5 according to the ACR BI-RADS 5th edition criteria. In cases of disagreement, the images were reviewed through discussion or adjudicated by a senior radiologist. For analysis, lesions were dichotomized: 4A and below were grouped as benign, while 4B and above were classified as malignant. This dichotomization was based on the BI-RADS 5th edition malignancy risk stratification, where 4A indicates low suspicion (2–10%) and 4B to 5 indicate progressively higher risk (> 10%) [18].

### Image segmentation

**Fig. 3:**
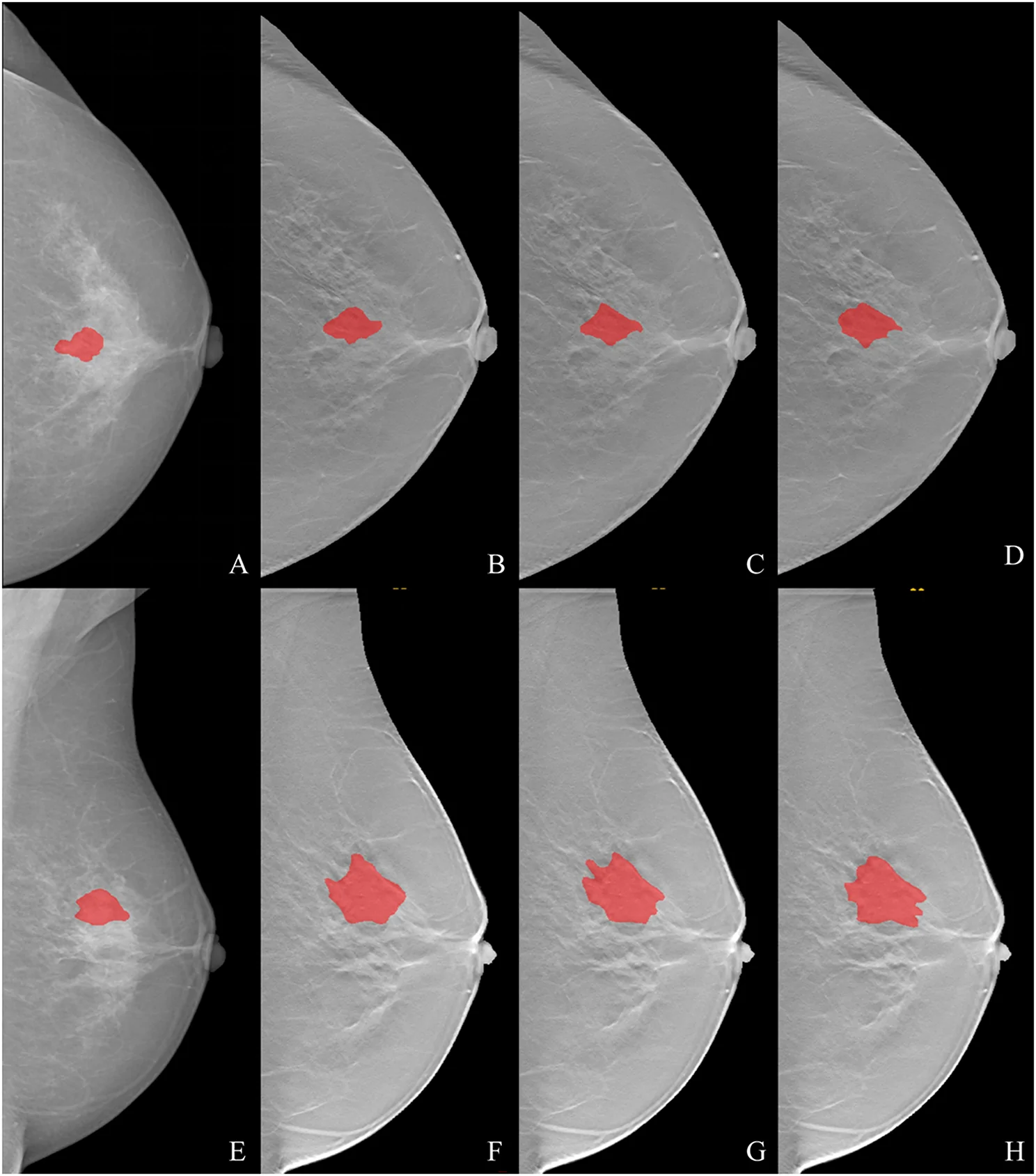
Representative diagram of ROI and VOI delineation for breast microcalcifications. This case involves a 58-year-old woman. Postoperative pathology confirmed DCIS in the left breast, and clustered amorphous microcalcifications were observed in the lesion. (**A**) ROI delineation on CC MG; (**B**-**D**) VOI delineation on partial slices of CC DBT; (**E**) ROI delineation on MLO MG; (**F**-**H**) VOI delineation on partial slices of MLO DBT

The two radiologists independently performed manual segmentation of all slices containing microcalcifications in both MG and DBT images using ITK-SNAP software (Version 3.8.0, www.itksnap.org). Both radiologists were blinded to the final pathological results. All regions of interest (ROIs) and volumes of interest (VOIs) were delineated around the calcification clusters. An example of the segmentation is shown in Fig. 3. To evaluate segmentation reproducibility, 30 cases (15 benign and 15 malignant) were randomly selected from the entire dataset. After a two-week interval, the same two radiologists independently repeated the segmentation of these cases. Intraclass correlation coefficients (ICCs) were calculated to assess intra-observer and inter-observer agreement. An ICC > 0.75 was considered to indicate good reproducibility. In cases of significant disagreement in ROI delineation, the final results were determined by consensus or adjudicated by a senior radiologist.

### Feature extraction

Radiomics feature extraction was performed using PyRadiomics. For MG and DBT images, manually delineated ROIs and VOIs were used for feature extraction, respectively. A comprehensive set of radiomics features was extracted. These included shape features, first-order statistics features, texture features, and higher-order features. Before feature extraction, DBT volumes were resampled to a uniform voxel spacing of 1 × 1 × 1 mm to reduce scale variations caused by differences in the original slice thickness.

Prior to being input into the DL framework, the ROI and VOI obtained from lesion delineation underwent standardized preprocessing. First, each image was cropped to retain lesion regions and eliminate interference from irrelevant surrounding tissues. Second, Z-score normalization was applied to the image intensity to standardize the gray-level distribution across different images. A ResNet-50 network pretrained on the ImageNet dataset was adopted as the backbone for DL classification. Using a transfer learning strategy, the network was appropriately fine-tuned to adapt to the characteristics of breast microcalcification images. During model training, a unified set of hyperparameters was employed for iterative optimization. The ResNet-50 model automatically learned high-level imaging representations of microcalcification regions through its multi-layer convolutional architecture, including texture features, gray-level distribution, and spatial structural information. The final classification layer generated the probability of lesion malignancy, which was defined as the DL score. This score was subsequently used for fusion modeling with radiomics features.

### Feature selection

Prior to radiomics feature selection, Z-score normalization was applied to standardize feature scales and reduce differences in value ranges. Features with ICC values > 0.75 were retained for subsequent statistical analysis. The feature selection process included the following steps. First, features with statistical significance were selected using an independent sample t-test (*P* < 0.05). Next, Pearson correlation analysis was conducted to evaluate linear correlations between features. Highly correlated features were considered redundant and were removed. Then, the maximum relevance and minimum redundancy (mRMR) algorithm was utilized to select features that were most relevant to the microcalcification diagnosis while reducing redundancy. Finally, the least absolute shrinkage and selection operator (LASSO) regression was utilized. The optimal regularization parameter λ was determined using ten-fold cross-validation. Features with non-zero coefficients were retained as the final selected features.

### Model construction

All models in this study were built using logistic regression (LR). The DL scores derived from the ResNet-50 architecture were integrated with radiomics features selected during the feature selection process to develop separate single-modality MG and DBT models. To further reduce feature dimensionality and avoid potential overfitting during feature fusion, all features from the single-modality models underwent a second round of LASSO-based feature selection. Subsequently, a combined MG-DBT model (MD model) and a BI-RADS model were constructed. For the BI-RADS model, binary BI-RADS labels were input into the logistic classifier. During model training, five-fold cross-validation was performed within the training set to evaluate model stability. The results were used as internal validation performance metrics.

### Statistical analysis

All statistical analyses and model construction were performed on the OmniMedAI (www.omnimai.com) and JiZhi Analytics (www.xsmartanalysis.com) platforms. Continuous variables were expressed as mean ± standard deviation (SD), and differences between groups were compared using independent samples t-tests. Categorical variables were presented as n (%), and group differences were evaluated using the Chi-square test or Fisher’s exact test. Receiver operating characteristic (ROC) curves were plotted to assess model performance. The DeLong test was used to compare differences in the area under the curve (AUC). Model performance was evaluated using accuracy, sensitivity, and specificity. Decision curve analysis (DCA) was performed to assess clinical utility. SHapley Additive exPlanations (SHAP) plots were used to illustrate feature importance. *P* < 0.05 was considered statistically significant.

## Results

### General characteristics

A total of 190 patients with pathologically confirmed breast microcalcifications were included in this study. Table 1 summarizes the baseline clinical characteristics of the training and test sets. No statistically significant differences were observed in any clinical variables between the two groups. The mean age was 48.01 ± 9.43 years in the training set and 49.35 ± 8.40 years in the test set.

**Table 1:** Baseline clinical characteristics of patients in the training and test sets

Variable		Training set (*n* = 133)	Test set (*n* = 57)	Statistic	*P*-value^1^
Age [mean ± SD]		48.01 ± 9.43	49.35 ± 8.40	-0.924	0.357
Menopausal status [n(%)]	No	80 (60.15)	31 (54.39)	0.546	0.460
	Yes	53 (39.85)	26 (45.61)		
Lesion location [n(%)]	Left	78 (58.65)	31 (54.39)	0.296	0.586
	Right	55 (41.35)	26 (45.61)		
Family history of breast cancer [n(%)]	No	126 (94.74)	56 (98.25)	1.218	0.270
	Yes	7 (5.26)	1 (1.75)		
Reproductive history [n(%)]	No	10 (7.52)	5 (8.77)	0.086	0.769
	Yes	123 (92.48)	52 (91.23)		
Breast tissue type [n(%)]	Fatty breast tissue	5 (3.76)	3 (5.26)	0.224	0.636
	Dense breast tissue	128 (96.24)	54 (94.74)		
Quadrant [n(%)]	Non-upper outer quadrant	36 (27.07)	20 (35.09)	1.235	0.267
	Upper outer quadrant	97 (72.93)	37 (64.91)		
BI-RADS [n(%)]	3	11 (8.27)	3 (5.26)	2.207	0.698
	4 A	59 (44.36)	23 (40.35)		
	4B	16 (12.03)	8 (14.04)		
	4 C	31 (23.31)	18 (31.58)		
	5	16 (12.03)	5 (8.77)		
Microcalcification morphology [n(%)]	Punctate	67 (50.38)	26 (45.61)	2.391	0.495
	Amorphous	29 (21.81)	10 (17.54)		
	Coarse heterogeneous	30 (22.56)	15 (26.32)		
	Fine pleomorphic	7 (5.26)	6 (10.53)		
Microcalcification distribution [n(%)]	Grouped	92 (69.17)	40 (70.18)	0.019	0.990
	Regional	17 (12.78)	7 (12.28)		
	Linear and segmental	24 (18.05)	10 (17.54)		
Histopathology [n(%)]	Benign	67 (50.38)	28 (49.12)	0.025	0.874
	Malignant	66 (49.62)	29 (50.88)		

No statistically significant differences were observed between the training and test sets in any clinical or imaging baseline characteristics

^1^ t-test, Fisher’s exact test, and Pearson’s Chi-square test

^2^ BI-RADS, Breast Imaging Reporting and Data System

### Feature extraction and selection

A total of 1,666 radiomics features were initially extracted from MG images. After sequential feature selection procedures, the seven most predictive features were ultimately retained. For DBT images, 2,394 radiomics features were extracted, and five optimal features were selected using the same procedures. Furthermore, DL scores of CC and MLO views for both MG and DBT images were computed based on the ResNet-50 framework for subsequent model construction.

### Construction of single modality models

**Fig. 4:**
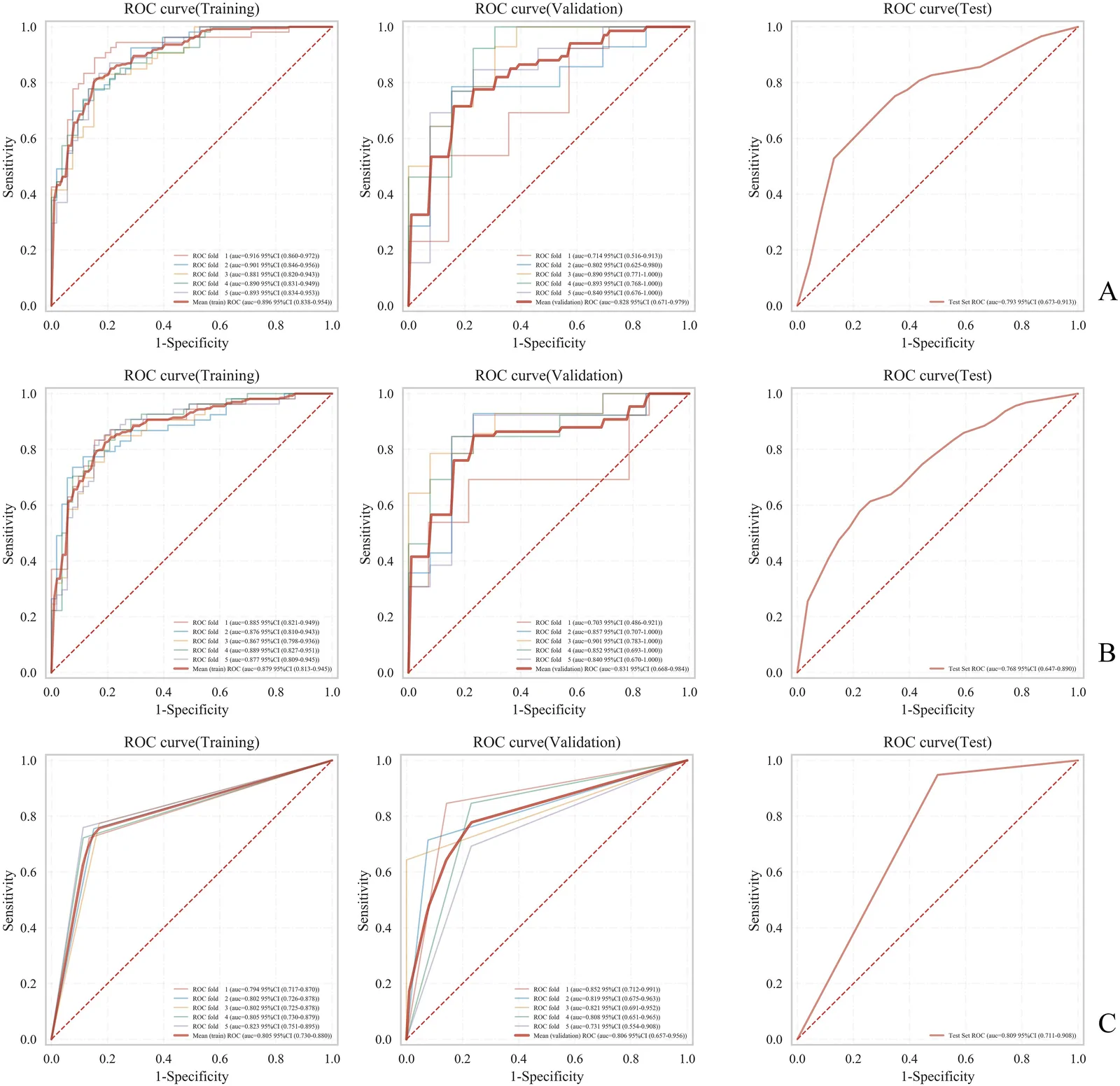
ROC curves of the three models in the training set, five-fold cross-validation, and test set. (**A**) MG model; (**B**) DBT model; (**C**) BI-RADS model

Single-modality prediction models were first built based on MG and DBT images. The MG model integrated the DL scores with selected radiomics features. This model achieved AUCs of 0.896, 0.828, and 0.793 in the training set, five-fold cross-validation, and test set, respectively. The DBT model was constructed using the same strategy and achieved AUCs of 0.879, 0.831, and 0.768 in the training set, five-fold cross-validation, and test set, respectively. For comparison, the BI-RADS model achieved AUCs of 0.805, 0.806, and 0.809 in the training, five-fold cross-validation, and test sets, respectively (Fig. 4).

### Construction of the integrated model

**Fig. 5:**
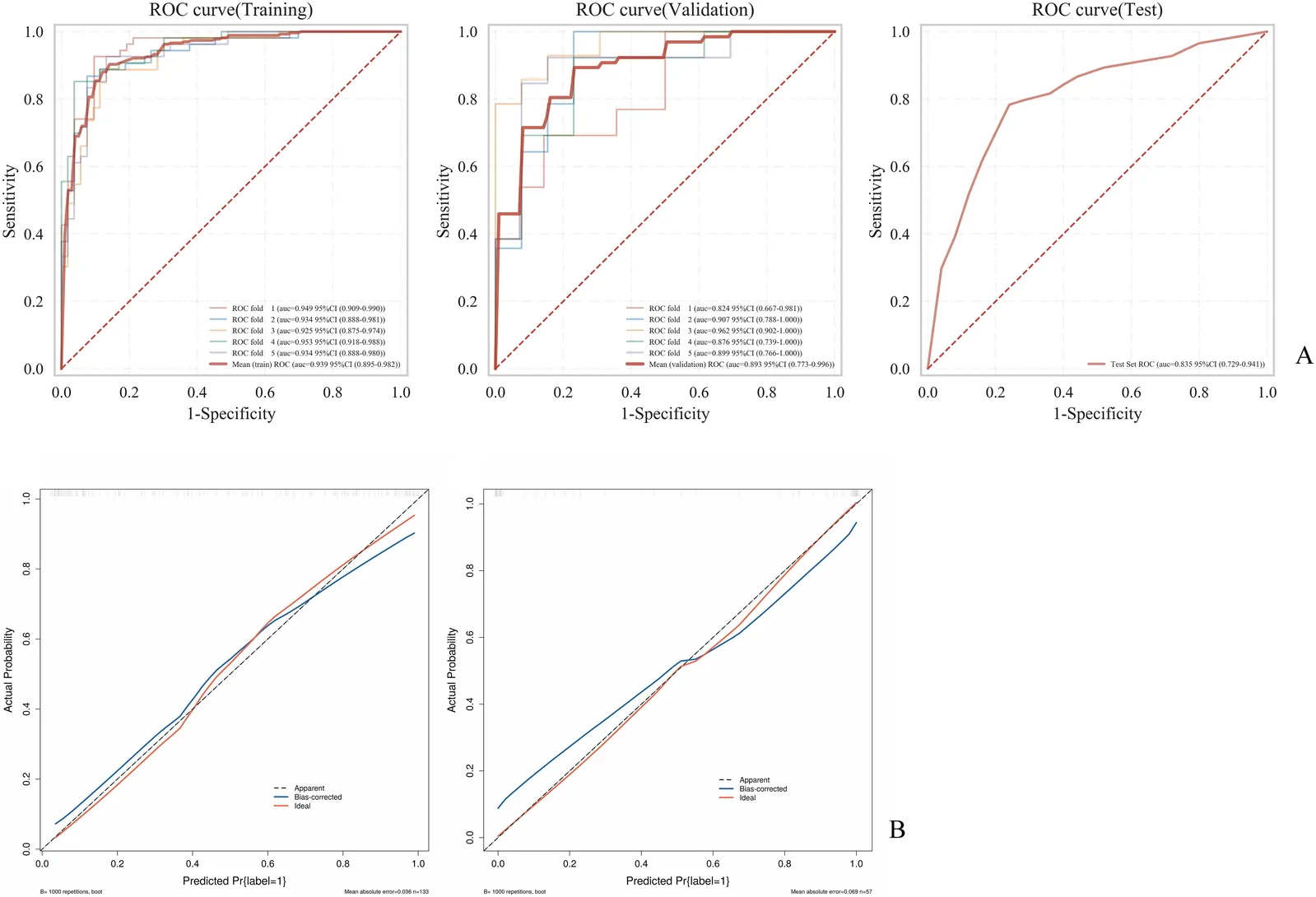
ROC curves and calibration curves of the MD model. (**A**) ROC curves in the training set, five-fold cross-validation and test set; (**B**) Calibration curves in the training and test sets

**Fig. 6:**
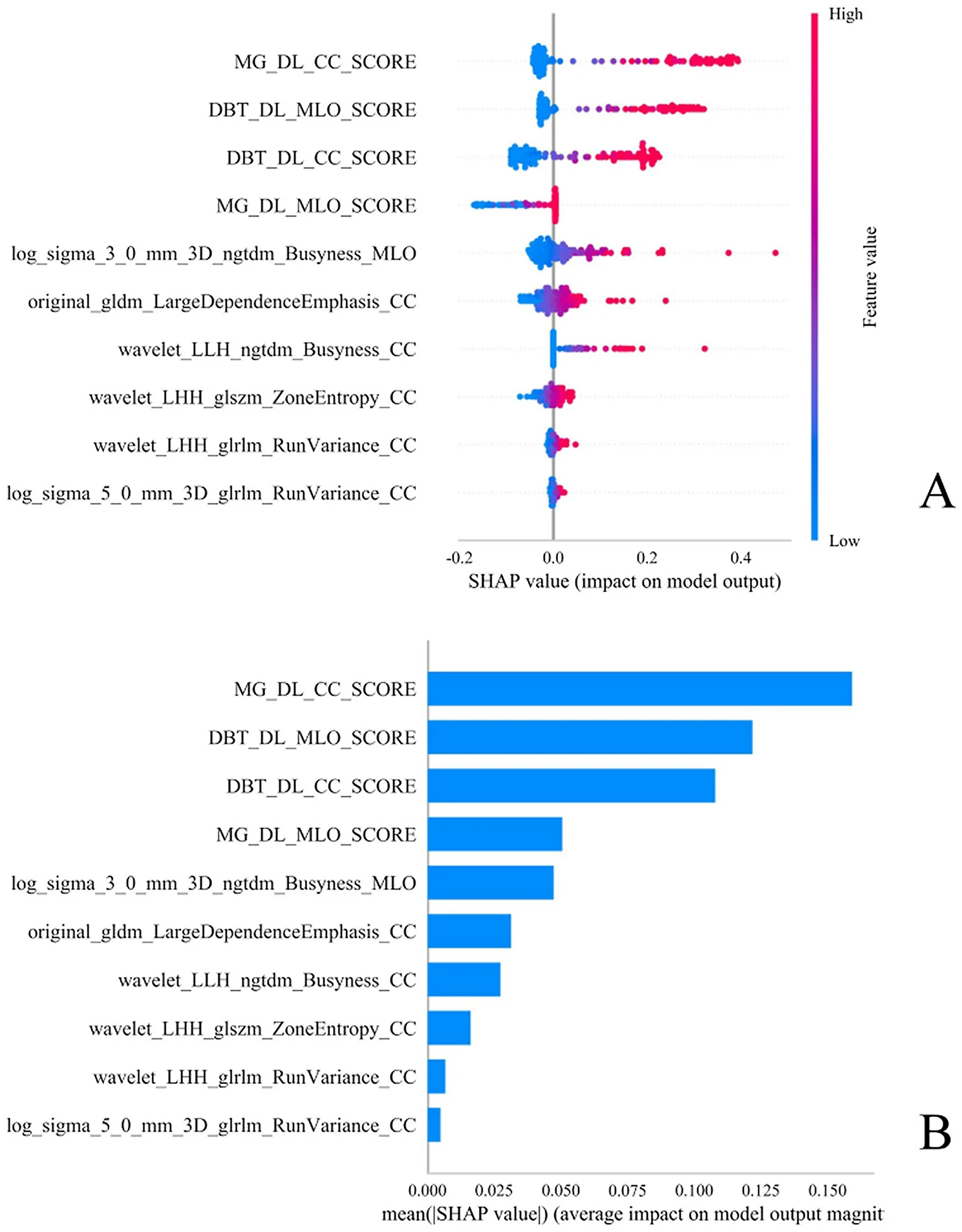
SHAP plots for the MD model. (**A**) Scatter plot; (**B**) Bar graph. The SHAP analysis revealed that the DL scores from the MG CC view and radiomics features reflecting textural complexity and spatial heterogeneity contributed the most to the model’s predictions

To integrate information from both MG and DBT images, the MD model was further developed. Radiomics features and DL scores from both modalities were fused for model construction. LASSO regression was used for further feature selection and to reduce feature redundancy. The final integrated model included four DL scores, three MG radiomics features, and three DBT radiomics features. The MD model showed excellent discriminatory performance. It achieved AUCs of 0.939, 0.893, and 0.835 in the training set, five-fold cross-validation, and test set, respectively (Fig. 5). Detailed performance metrics are provided in Table 2. The calibration curve of the training set closely approximated the ideal reference line, whereas the test set exhibited a slight deviation. The Brier scores were 0.112 and 0.161 for the training and test sets, respectively, suggesting that the model maintained good calibration despite a modest decline in the test set. Furthermore, SHAP analysis (Fig. 6) was performed to rank the importance of the included features.

**Table 2:** Comparison of diagnostic performance among the four models

Model	Dataset type	AUC (95%CI)	Accuracy (%)	Sensitivity (%)	Specificity (%)
MG model	Training	0.896 (0.838–0.954)	83.5	84.3	82.6
	Five-fold cross-validation	0.828 (0.671–0.979)	76.8	76.2	77.1
	Test	0.793 (0.673–0.913)	71.9	75.0	69.0
DBT model	Training	0.879 (0.813–0.945)	83.1	81.3	84.8
	Five-fold cross-validation	0.831 (0.668–0.984)	78.3	77.5	78.7
	Test	0.768 (0.647–0.890)	68.4	53.6	82.8
MD model	Training	0.939 (0.895–0.982)	89.8	89.2	90.5
	Five-fold cross-validation	0.893 (0.773–0.996)	82.0	80.5	83.3
	Test	0.835 (0.729–0.941)	77.2	67.9	86.2
BI-RADS model	Training	0.805 (0.730–0.880)	80.4	74.6	86.4
	Five-fold cross-validation	0.806 (0.657–0.956)	80.4	74.8	86.4
	Test	0.809 (0.711–0.908)	80.7	92.9	69.0

Five-fold cross-validation was performed within the training set to evaluate model stability. The results were used as internal validation performance metrics

^1^ MG, mammography

^2^ DBT, digital breast tomosynthesis

^3^ BI-RADS, breast imaging reporting and data system

^4^ AUC, area under the curve

^5^ CI, confidence interval

### Model comparison and clinical utility validation

**Fig. 7:**
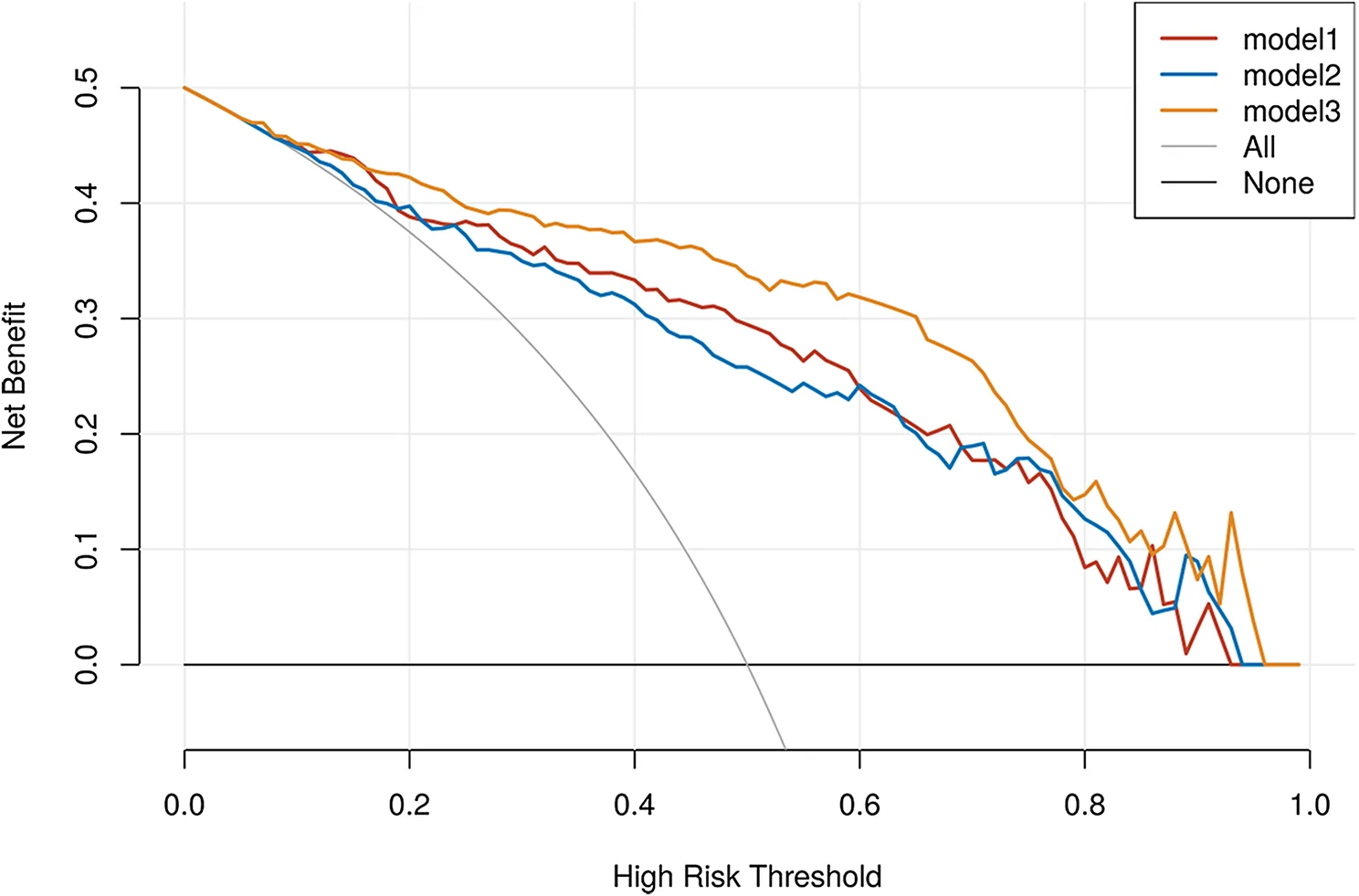
DCA curve analysis for the MG model (model 1), DBT model (model 2), and MD model (model 3)

**Fig. 8:**
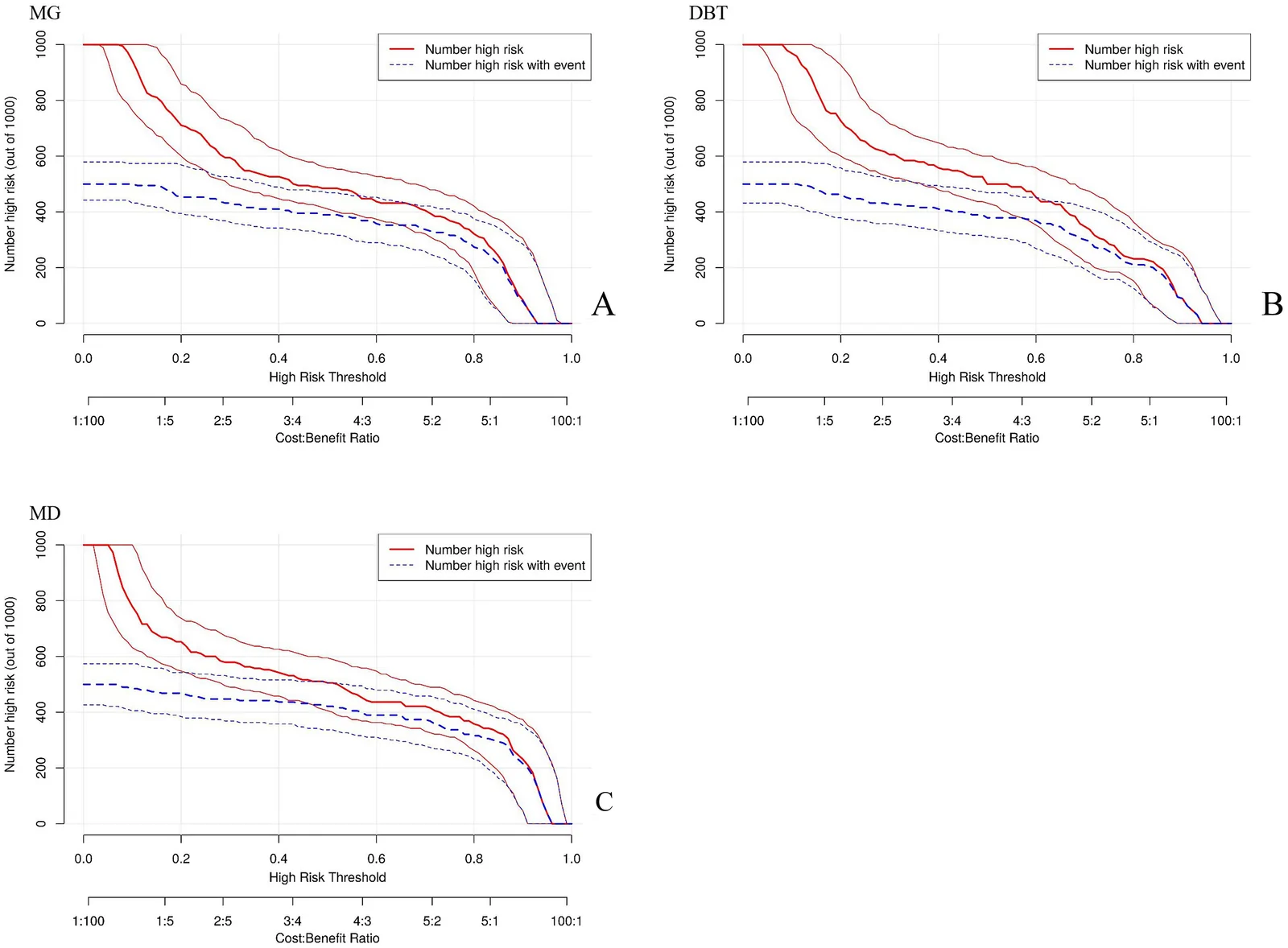
CIC for the three models. (**A**) MG model; (**B**) DBT model; (**C**) MD model

To evaluate the clinical utility of the models, their performance was compared with that of a BI-RADS model based on the BI-RADS assessments assigned by radiologists. The DeLong test results (Table 3) showed that the AUC of the MD model was significantly higher than those of the MG model, DBT model, and BI-RADS model (*P* < 0.05). No statistically significant differences were found in the other pairwise comparisons. DCA showed that, within the threshold probability range of 0.1 to 0.9, the MG, DBT, and MD models provided greater net benefits than the treat-all and treat-none strategies (Fig. 7). Notably, within the range of 0.1 to 0.75, the MD model provided the highest clinical net benefit. This finding indicated its superior value in supporting clinical decision-making. Furthermore, the clinical impact curve (CIC) showed that when the risk threshold exceeded 0.2, the MD model achieved an optimal balance between identifying true positives and controlling false positives (Fig. 8).

**Table 3:** DeLong test *P*-values for the MG model, DBT model, MD model, and BI-RADS model

Model	MG model	DBT model	MD model	BI-RADS model
MG model		0.604	0.022*	0.121
DBT model	0.604		0.005*	0.322
MD model	0.022*	0.005*		0.005*
BI-RADS model	0.121	0.322	0.005*	

*P < 0.05, statistically significant difference

^1^MG, mammography

^2^DBT, digital breast tomosynthesis

^3^BI-RADS, breast imaging reporting and data system

## Discussion

It is noteworthy that most previous breast imaging studies reported that single DBT models outperformed single MG models. For instance, Niu et al. [19] developed radiomics models and reported a training AUC of 0.850 for DBT, which was markedly higher than the AUC of 0.727 for MG. However, their study mainly included mass-type lesions and included few early breast lesions presenting with microcalcifications. Population-based screening research by Lång et al. [20] also revealed inherent drawbacks of DBT. DBT tends to overdetect benign stellate distortions. This may introduce false positives and disturb quantitative texture analysis. In contrast, MG may provide more stable visualization of tiny microcalcification features. In this study, we compared single-modality radiomics-deep learning models based on MG and DBT images. The DBT model yielded a slightly lower AUC than the MG model. However, the DeLong test showed no statistically significant difference between the two models. This finding may be partially explained by the controlled trial from Clauser et al. [21]. Wide-angle DBT uses oblique X-ray beams and may generate reconstruction artifacts. These factors can blur microcalcification margins and reduce the signal-to-noise ratio of calcification-related texture features. As a result, the ability of extracted features to distinguish benign from malignant lesions may be weakened. This result may be related to the characteristics of our study cohort. All suspicious lesions in our cohort were primarily characterized by microcalcifications. This study population may have magnified the inherent limitations of DBT in microcalcification depiction, leading to slightly inferior diagnostic performance of the DBT model.

Given the respective limitations of standalone MG and DBT models, multimodal fusion has emerged as a feasible strategy to offset individual imaging weaknesses. Consistent with the findings reported by Niu et al. [19] and Peng et al. [22], our combined model integrated radiomics features and DL scores from MG and DBT images. It showed better performance in differentiating benign from malignant breast microcalcifications than either of the single-modality models. The DeLong test further confirmed that the MD model performed significantly better than the single-modality models. Most existing MG-DBT multimodal studies have focused mainly on breast masses. Few studies have specifically investigated lesions presenting primarily as suspicious microcalcifications. Our study focused on microcalcification lesions and provided supportive evidence for multimodal fusion in this less-studied clinical subgroup. The improved performance of the dual-modality model may be partly explained by the complementary imaging information captured by MG and DBT. MG can clearly delineate microcalcification margins. This may partly compensate for the reduced signal-to-noise ratio of calcification textures induced by reconstruction artifacts and oblique X-ray noise in wide-angle DBT. In contrast, DBT can reduce the effect of overlapping glandular tissue and reveal spatial lesion patterns that are not easily observed on MG images. Therefore, the combination of 2D and 3D imaging may support more comprehensive lesion characterization. In addition to cross-modality integration, the joint use of two feature categories may also improve model performance. Marathe et al. [23] constructed a LightGBM classifier using only handcrafted radiomics signatures to predict the malignancy of amorphous calcifications in 276 patients. Their model achieved 100% sensitivity but only 35% specificity, which may increase the risk of false-positive results. This finding suggests potential diagnostic limitations when relying on radiomics features alone. Guo et al. [24] developed a multimodal model combining mammography and MRI data. By integrating radiomics and DL features to predict breast cancer lymph node metastasis, their model achieved a test set AUC of 0.846. This value was higher than that of models using only radiomics features (AUC = 0.756) or DL alone (AUC = 0.712). Our results are consistent with this observation. We combined interpretable radiomics features with high-level abstract DL scores to balance clinical explainability and the ability to capture subtle image patterns. The synergy between the two feature sets may mitigate the diagnostic limitations of single-feature models and provide relatively stable quantitative references for distinguishing benign and malignant breast microcalcifications.

We performed SHAP analysis to visualize the contribution of each feature. This analysis helped interpret the decision-making process of the integrated model and improved transparency for potential clinical application [25, 26]. In this study, the SHAP analysis showed that the DL scores from the MG CC view made the greatest contribution. This is likely because the CC view compresses breast tissue more uniformly within the imaging plane. It may also include lesions in the retromammary and central regions more completely. In addition, the CC view provides a direct visualization of the distribution and morphology of microcalcification clusters. It may also reduce the overlap from axillary tail tissue that can occur in the MLO view, resulting in a more focused field of view. This observation is consistent with the findings of Nana et al. [27]. As a 2D imaging technique, MG has an inherent advantage in showing the morphology and sharpness of microcalcifications. The DL scores from the DBT MLO view had the second-highest contribution. The tomosynthesis characteristics of DBT can reduce tissue superposition artifacts inherent in this projection view. In addition, the 3D imaging capability of DBT provides information about the spatial relationship between the microcalcification clusters and anatomical structures, such as the pectoral muscle, skin, and nipple. This information may help the model better evaluate lesion extent and spatial localization.

Among the radiomics features, the texture feature “log_sigma_3_0_mm_3D_ngtdm_Busyness_MLO” showed a relatively high contribution. Higher values of this feature were positively associated with malignant risk. This feature quantifies the textural complexity of the image region. It captures micro-level irregularity and complexity, which may be consistent with the morphological characteristics of “fine pleomorphism” and “heterogeneity” commonly observed in malignant microcalcification clusters [10, 28]. Among the wavelet-transformed features, “wavelet_LLH_ngtdm_Busyness_CC” and “wavelet_LHH_glszm_ZoneEntropy_CC” also showed positive contributions to the prediction of malignancy. Higher values of the former may indicate increased texture complexity of malignant calcifications at specific frequencies. In contrast, higher values of the latter reflect greater randomness in pixel distribution within the malignant lesion region. We hypothesize that these imaging features may be associated with the disordered proliferation of malignant tumor cells [29]. Further validation using proliferation-related histopathological markers, such as Ki-67, is required to systematically illustrate the potential biological basis of these imaging signatures. Regarding run-length features, both “wavelet_LHH_glrlm_RunVariance_CC” and “log_sigma_5_0_mm_3D_glrlm_RunVariance_CC” showed positive contributions to malignancy prediction. Higher values of these features indicate greater variability in the run length. This finding further supports the heterogeneity of malignant lesions in terms of spatial distribution.

Based on the results of model construction and feature analysis, we further compared the multimodal fusion model with a BI-RADS-based diagnostic model. The BI-RADS classification may rely heavily on the subjective visual assessment and clinical experience. Its diagnostic consistency may be affected by differences in physician experience [30]. In contrast, the MD model automatically extracted and selected the most discriminative features from large-scale quantitative data. This process may reduce diagnostic variability caused by intra-observer and inter-observer differences. These results suggest that AI-based image analysis has the potential to improve diagnostic consistency [31, 32].

Finally, the potential clinical value of this model was further explored beyond the reduction of unnecessary invasive biopsies. First, the model may serve as a screening aid in breast imaging settings. It can identify lesions with elevated malignant risk and provide supportive information for radiologists. This may improve screening efficiency and reduce the risk of missed diagnoses. Second, primary care and community hospitals often lack experienced breast radiologists. This model may provide quantitative evidence to support clinical decision-making and help reduce diagnostic disparities between different healthcare settings. Third, the model may assist junior radiologists and residents during routine image interpretation. It can improve their ability to recognize suspicious lesions and reduce diagnostic errors. Fourth, the model has the potential to be integrated into standard breast imaging workstations. It enables semi-automatic image analysis while fitting into existing reading workflows. This may improve overall efficiency and facilitate clinical decision-making.

### Limitations and future directions

This study was a single-center retrospective analysis with a limited sample size. Cases with additional imaging findings were excluded during screening, which may lead to selection bias. In addition, no external validation was conducted, so the generalization of the model needs further validation with multicenter data. For lesion stratification, BI-RADS 4A and 4B were used as the threshold to distinguish benign from malignant lesions. However, BI-RADS 4A lesions still carry a 2%-10% risk of malignancy, which may reduce the precision of risk stratification. In terms of imaging, DBT images contain inherent noise, which may impair the stability and repeatability of some radiomics features. For multimodal feature fusion, only simple feature concatenation was used. This approach could not achieve adaptive dynamic weight assignment for MG and DBT features. Future studies will explore advanced multimodal fusion algorithms to further optimize the diagnostic performance of the integrated model.

## Conclusions

In conclusion, this exploratory study developed a multimodal prediction model integrating radiomics features and DL scores from MG and DBT images. The preliminary results suggested that the model achieved good performance in differentiating benign and malignant lesions and showed better performance than conventional BI-RADS classification. This approach may provide a non-invasive quantitative tool to evaluate microcalcification clusters before surgery. It might help clinicians optimize decision-making, reduce unnecessary invasive biopsies, and improve diagnostic consistency.

## Data Availability

The datasets used and analysed during the current study are available from the corresponding author on reasonable request.

## Abbreviations

AUC: Area under the curve
BI-RADS: Breast imaging reporting and data system
CC: Craniocaudal
DBT: Digital breast tomosynthesis
DCA: Decision curve analysis
ICC: Intraclass correlation coefficient
LASSO: Least absolute shrinkage and selection operator
LR: Logistic regression
MG: Mammography
MLO: Mediolateral oblique
mRMR: maximum relevance and minimum redundancy
ROC: Receiver operating characteristic
SHAP: SHapley additive explanations
VOI: Volume of interest
